# Assessment of the face validity of two pain scales in Kenya: a validation study using cognitive interviewing

**DOI:** 10.1186/1472-684X-11-5

**Published:** 2012-07-10

**Authors:** Kristin TL Huang, Claudio Owino, Rachel C Vreeman, Mildred Hagembe, Festus Njuguna, R Matthew Strother, Gregory P Gramelspacher

**Affiliations:** 1 USAID - Academic Model Providing Access to Healthcare (AMPATH), P.O. Box 4806, Eldoret, Kenya; 2 Harvard Medical School, 25 Shattuck Street, Boston, Massachusetts, 02115, USA; 3 Moi University School of Medicine, P.O. Box 4606, 030100, Eldoret, Kenya; 4 Department of Pediatrics, Indiana University School of Medicine, 410 W. 10th Street, HITS 1000, Indianapolis, Indiana, 46202, USA; 5 Moi Teaching and Referral Hospital, Nandi Road, P.O. Box 3, 30100, Eldoret, Kenya; 6 Department of Medicine, Indiana University School of Medicine, 545 Barnhill Drive, E317, Indianapolis, Indiana, 46202, USA

## Abstract

**Background:**

Patients in sub-Saharan Africa commonly experience pain, which often is un-assessed and undertreated. One hindrance to routine pain assessment in these settings is the lack of a single-item pain rating scale validated for the particular context. The goal of this study was to examine the face validity and cultural acceptability of two single-item pain scales, the Numerical Rating Scale (NRS) and the Faces Pain Scale-Revised (FPS-R), in a population of patients on the medical, surgical, and pediatric wards of Moi Teaching and Referral Hospital in Kenya.

**Methods:**

Swahili versions of the NRS and FPS-R were developed by standard translation and back-translation. Cognitive interviews were performed with 15 patients at Moi Teaching and Referral Hospital in Eldoret, Kenya. Interview transcripts were analyzed on a question-by-question basis to identify major themes revealed through the cognitive interviewing process and to uncover any significant problems participants encountered with understanding and using the pain scales.

**Results:**

Cognitive interview analysis demonstrated that participants had good comprehension of both the NRS and the FPS-R and showed rational decision-making processes in choosing their responses. Participants felt that both scales were easy to use. The FPS-R was preferred almost unanimously to the NRS.

**Conclusions:**

The face validity and acceptability of the Swahili versions of the NRS and FPS-R has been demonstrated for use in Kenyan patients. The broader application of these scales should be evaluated and may benefit patients who currently suffer from pain.

## Background

Sub-Saharan Africa bears a disproportionate measure of the global burden of many diseases, as well as their attendant morbidities, including pain [1]. The prevalence of pain is amplified by lack of access to health facilities, late presentation, inadequate diagnosis, treatment unavailability, lack of medical education regarding pain control, and scarcity and underprescribing of opioids [1-4].

Pain in sub-Saharan Africa has been studied primarily in three patient populations: HIV/AIDS patients, cancer patients, and palliative care patients. The prevalence of pain in African patients with HIV ranges from 59 to 98%, depending on disease stage, similar to the pain prevalence found in other HIV patient populations [5-8]. Cancer, increasingly prevalent in sub-Saharan Africa with rates of cancer expected to quadruple over the next 50 years, is also frequently associated with pain [9,10]. One study conducted in Uganda and South Africa found the prevalence of pain was 87.5% in cancer patients attending palliative care services [11]. Patients at the ends of their lives are particularly vulnerable to pain. A study of hospice patients in Uganda found that two-thirds had experienced severe, prolonged pain before having their pain adequately treated [1]. In Kenya, researchers have found that patients at the ends of their lives often die in pain [12,13].

**Figure 1:**
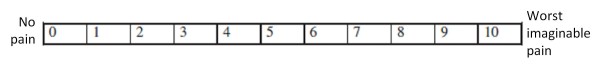
**Numerical Rating Scale.** Instructions for patients: “If 0 means ‘no pain,’ and 10 means ‘the worst pain that you can imagine,’ on this scale from 0 to 10, what is your current level of pain?”

**Figure 2:**
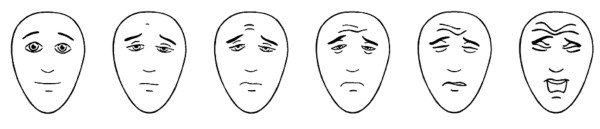
**Faces Pain Scale-Revised.** Instructions for patients: “These faces show how much something can hurt. The face on the left shows no pain. The faces show more and more pain proceeding from left to right, up to the face on the right – it shows the most pain. Point to the face that shows how much you hurt right now.” Scored 0-2-4-6-8-10 This figure has been reproduced with permission of the International Association for the Study of Pain® (IASP®). The figure may not be reproduced for any other purpose without permission.

In order to treat pain effectively, clinicians must assess whether patients are experiencing pain, manage the pain appropriately, and then reassess whether efforts to relieve the pain have been successful. Single-item, continuous rating scales, commonly used to assess pain, are a valuable tool for clinicians to ascertain pain in patients [14,15]. These include the visual analog scale (VAS), the graphic rating scale (GRS), the numerical rating scale (NRS), and the verbal rating scale (VRS). Studies comparing these scales have found similar accuracy and validity among scales, though the Numerical Rating Scale (NRS) (Figure 1) has been found to have the highest sensitivity combined with simplicity of administration [16-22]. A similar scale, the Faces Pain Scale, developed for use in pediatric populations, but now validated in all age ranges, may be particularly helpful in patients who are illiterate or have language difficulties [23-27]. The Faces Pain Scale-Revised (FPS-R) (Figure 2) can be scored along the same 0 to 10 metric as the NRS, and has been shown to have the best psychometric properties in school-aged children (4 to 12 years) compared to several other faces scales, including the Oucher photographic scale and the Wong-Baker Faces Pain Scale, as well as the Pieces of Hurt Tool and the VAS [24,28,29].

No single-item pain measurement tool, including the FPS-R and the NRS, has been validated for use in East Africa. This is significant as not all pain measurement tools maintain their reliability when translated into another language or used in another culture [14]. In sub-Saharan Africa, there have been a limited number of studies examining the cross-cultural validity of pain assessment tools. The VAS and VRS were administered to a cohort of 100 Nigerian patients, and both scales were found to have high levels of correlation [30]. A study of a six-graded faces pain scale in children ages 4–12 in South Africa compared the scale to a designated nurse’s assessment of the child’s pain. This study found a strong correlation between the two methods and confirmed the validity of using a faces pain scale in this population [31]. Another study undertaken in Nigeria found that the VAS, NRS, and Oucher photographic faces pain scales demonstrated high convergent validity in Nigerian children [32]. Correlation and convergent validity are important components of validating a scale for cross-cultural use, but these measures may not express how well concepts are understood or questions are accepted in a particular context. Promisingly, a pilot acceptability study of the FPS-R showed that 20 adult Ugandan hospice patients were easily able to choose a response to the scale, but did not further describe the participants’ understanding of the scale [33].

There is a pressing need for cross-cultural validation of functional and appropriate pain assessment tools for use in East Africa. Recently, particular attention has been paid to Kenya regarding its failure to provide satisfactory pain assessment and control in children [34]. Clinicians in Kenya are not trained to assess pain, pain assessment is not frequently performed in hospitals, and pain is often undertreated due to fear of opioids and lack of prioritization of pain relief [34]. Assessing pain accurately and in a culturally acceptable manner are crucial first steps to combating these pain management challenges, both for pediatric and adult patients. In this study, we chose to examine the face validity and cross-cultural acceptability of the NRS and the FPS-R in adult and pediatric Kenyan patients.

## Methods

### Setting

This study was performed at Moi Teaching and Referral Hospital (MTRH) in Eldoret, Kenya. MTRH is a national referral hospital with a catchment area of Nyanza Province, North Rift Valley Province, and Western Province, which have a joint population of about 11.24 million people. The hospital currently accommodates 500 inpatient beds and a large outpatient department. Over the past six years, MTRH has been building an oncology program and recently established palliative care under the auspices of the oncology department.

This study was approved by the Institutional Research and Ethics Committee of Moi University School of Medicine and by the Institutional Review Board of Indiana University.

### Development of the Swahili versions of FPS-R and NRS

**Figure 3:**
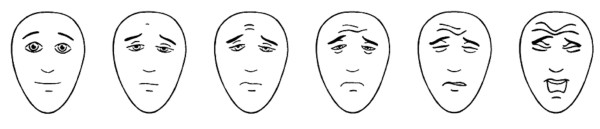
**Faces Pain Scale-Revised – Swahili.** Instructions for patients: “Hizi nyuso zaonyesha jinsi kitu kinaweza umiza. Uso ulio kushoto hauonyeshi uchungu. Nyuso hizo zaonyesha uchungu zaidi na zaidi kuanzia kushoto kuelekea kulia hadi uso ulio kulia – inaonyesha uchungu ulio mwingi zaidi. Lenga uso unaoonyesha jinsi unavyoumia sasa hivi.” This figure has been reproduced with permission of the International Association for the Study of Pain® (IASP®). The figure may not be reproduced for any other purpose without permission.

**Figure 4:**
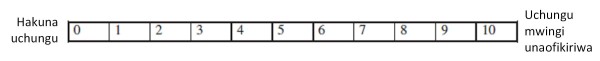
**Numerical Rating Scale – Swahili.** Instructions for patients: “Ikiwa 0 inamaanisha ‘hakuna uchungu,’ na 10 inamaanisha ‘uchungu mbaya zaidi wenye unaweza kufikiria,’ katika mizani hii ya 0 mpaka 10, ni kipi kiwango chako cha sasa cha uchungu?”

English and Swahili are the two national languages of Kenya. An experienced translator skilled in both Swahili and English translated the FPS-R and NRS into Swahili. The scales were independently back-translated into English by bilingual study staff without prior exposure to the original English-language versions. This process of translation and back-translation was repeated until the scales were consistent in both languages (Figure 3 and Figure 4). The translations were reviewed for accuracy by a bilingual committee, which would have discussed any challenges or terms over which there were disagreement; however, no such conflicts arose.

### Population

Using an online random number generator corresponding to inpatient bed numbers, we randomly selected five adult medical, five adult surgical, and five pediatric inpatients at MTRH. These patient populations were chosen to ensure inclusion of a broad range of disease states and age ranges. Furthermore, the medical, surgical, and pediatric wards are the largest and most representative wards at MTRH, and were prioritized for inclusion in this study by our multispecialty team of pediatricians, internists, oncologists, and surgeons. We included patients ages 8 years and over, with the ability to speak English or Swahili, and with the mental and physical capacity to give informed assent or consent and respond to the question probes. Verbal informed consent and/or assent were obtained from all participants. We recruited patients until thematic saturation was reached at 15 interviews, which corresponds to the literature that states performing 12–15 cognitive interviews is usually sufficient to maximize the yield of useful information [35].

### Cognitive interviewing and data collection

The two pain scales were administered to each participant. Subsequently, cognitive interviewing, a qualitative research method, was used to examine how participants understand, process, and respond to the pain scales [35]. Cognitive interviewing has become a key technique in uncovering potential problems with survey questionnaires through a process of administering draft survey questions and then probing how subjects comprehend, recall, decide, and respond to the questions [36]. Use of cognitive interviewing in the pediatric population has been well-documented and has proven to be a valuable tool for elucidating concerns specific to children [37-39].

Four trained cognitive interviewers performed the interviews and were assisted at all times by the first author to ensure consistency. Standardized, concurrent, proactive question probes were used as documented in Additional file 1: Appendix. A combination of comprehension/interpretation probes (e.g. “In this question, what does the word ‘pain’ mean to you?” and “Which face would you choose if you were not experiencing any pain?”), as well as general probes (e.g. “How did you decide your answer to this question?”) were employed. Eight participants were shown the FPS-R first, along with its corresponding cognitive interview questions, while seven participants were shown the NRS first, so as to lessen the effect of order on preference.

Demographic and clinical data were also gathered for each patient from chart review. These data included age, date of hospital admission, working diagnosis, chronic medical problems, and any pain medications received.

### Data analysis

The interviews were audiotaped and transcribed. Each transcript was then summarized on a question-by-question basis. Results across the interviews were compiled to create an organized testing report for each cognitive interviewing probe that included the major themes elicited by the probe and the number of respondents whose answers touched on those themes. Any minor but significant responses were also highlighted in the testing report. The testing report was analyzed independently by three investigators to seek out problems encountered repeatedly across interviews and whether reported problems could reasonably be attributed to the characteristics of the pain tools themselves [34]. Any discrepancies were discussed by the investigators until agreement was reached. The conclusions of this analysis were then applied to the FPS-R and NRS to determine if any changes needed to be made based on the results of the cognitive interviewing process.

## Results

### Participant characteristics

Fifteen interviews were performed, including seven women and nine men, with an age range of 8 to 69 years. One subject was excluded from analysis because his diagnosis of acute psychosis rendered him unable to adequately answer questions. Table 1 describes the participant characteristics, including admission diagnosis. Responses to the cognitive interviewing probes were easily elicited and participants did not demonstrate or express any difficulty in answering them.

**Table 1:** Summary of subjects

*Subject*	*Age (years)*	*Gender*	*Service*	*Diagnosis*
1	11	Male	Pediatrics	Heart failure in rheumatic heart disease
2	8	Male	Pediatrics	Pulmonary tuberculosis
3*	10	Male	Pediatrics	Acute psychosis
4	58	Female	Surgery	Mesenteric tumor
5	34	Female	Surgery	Breast cancer
6	43	Male	Medicine	Cryptococcal meningitis
7	35	Male	Medicine	Unspecified connective tissue disease
8	9	Male	Pediatrics	Gastritis or peptic ulcer disease
9	9	Male	Pediatrics	Nephrotic syndrome
10	30	Female	Surgery	Paraplegia and spinal cord injury from motor vehicle accident
11	27	Female	Surgery	Necrotizing fasciitis
12	69	Male	Medicine	Liver mass with obstructive jaundice
13	12	Female	Pediatrics	Congestive heart failure in rheumatic heart disease
14	43	Female	Medicine	Peptic ulcer disease
15	36	Female	Medicine	Chronic idiopathic thrombocytopenic purpura
16	48	Male	Surgery	Esophageal cancer

*Subject 3 was excluded from analysis due to his diagnosis of acute psychosis.

### Faces pain scale-revised (FPS-R)

**Figure 5:**
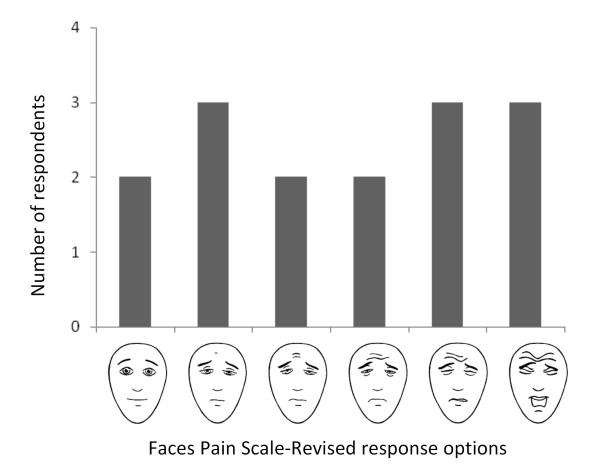
**FPS-R Responses. **The y-axis depicts the number of participants who chose each face to represent their pain

The responses to the FPS-R ranged widely, from the first face (no pain) to the last face (the worst pain of your life) (Figure 5).

#### Preference

The FPS-R was virtually universally preferred to the Numerical Rating Scale, as 14 of 15 participants favored the FPS-R, and the remaining participant stated that he liked both scales. Participants found that the faces were easy to understand as they visibly depicted pain and the absence of pain. They felt that the illustrated expressions made it easier to choose a response to the pain scale, and that the faces make it easier for medical personnel to understand the pain that a patient is in: “[Using the faces pain scale] would help because when the doctor or nurse sees the expression on the face, they will know the pain I’m feeling. The faces are easier for both the patient and the doctor” (Subject 5; 35 year-old woman with breast cancer).

#### Decision-making process

Twelve out of 14 participants thought that it was easy to choose a response on the FPS-R, while one was unsure and one thought it was difficult because she didn’t have any pain. The reasoning process the participants used included comparing their current pain to prior levels of pain during the same hospitalization: “Yesterday, they did a lumbar puncture – before that procedure, it was very painful, but today it’s not as bad” (Subject 6; 43 year-old man with cryptococcal meningitis). They also compared the facial expressions to their own perceived pain level: “My pain doesn’t reach to face four, just to face three” (Subject 14; 43 year-old woman with peptic ulcer disease). “I chose the first one because according to me, I don’t have pain, but the other faces show pain” (Subject 15; 36 year-old woman with chronic idiopathic thrombocytopenic purpura). “The others don’t have a lot of pain, but I chose the sixth face because I have a lot of pain” (Subject 13; 12 year-old girl with congestive heart failure in rheumatic heart disease). Eleven of 12 participants thought that facial expressions are a fair reflection of pain (one participant was unsure).

#### Comprehension

The participants were able to accurately describe what the progression of faces meant on the scale: “The first one shows no pain, the second shows a little pain, the third shows more pain, the fourth is much pain, the fifth is a lot a lot of pain, the sixth is very very painful” (Subject 5). Fourteen out of 15 participants were able to correctly identify the first face as the face they would choose if they were not experiencing any pain, with one participant choosing the second face. All 15 participants correctly identified the last face as the face they would choose if they were experiencing the worst pain of their lives.

#### Ease of use

Ten out of 14 participants felt that nothing needed to be changed to make the scale easier to understand. One boy suggested putting ears on the faces (Subject 1, 11 year-old boy with congestive heart failure in rheumatic heart disease). Two men stated that the wrinkles on the faces help show pain. One woman stated that we should “give [the faces] medicine to make them feel better” (Subject 11; 27 year-old woman with necrotizing fasciitis). Ten out of 10 participants felt that there was nothing confusing about the FPS-R.

The cognitive interviewing process supported that the FPS-R is acceptable to patients as a mechanism for assessing pain and represents their pain appropriately.

### Numerical rating scale (NRS)

**Figure 6:**
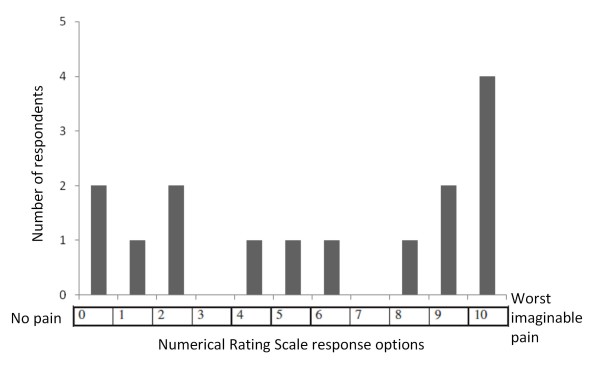
**Numerical Rating Scale Responses. **The y-axis represents the number of participants who chose each number to represent their pain

There were a wide variety of responses to the NRS, ranging from zero to 10 (Figure 6). While the FPS-R was preferred to the NRS, participants generally felt that the NRS would still be helpful in explaining their pain to their health care providers.

#### Decision-making process

Eleven of 14 participants thought that it was easy to arrive at an answer, while one was unsure. Two participants felt that it was difficult, one because she “had to think first” (Subject 15), and one because “you have to understand yourself and the progress of your pain before you can answer according to the scale” (Subject 10; 30 year-old woman with paraplegia and spinal cord injury from a motor vehicle accident). Similar to the reasoning process that some participants employed in choosing a response to the FPS-R, some patients chose their response to the NRS by comparing their current pain to prior pain during the hospitalization: “It’s not like yesterday, today is better” (Subject 6). “My pain is improving; it is not as bad as when I came in” (Subject 14).

#### Comprehension

The participants were able to accurately describe what the progression of the numbers from 0 to 10 meant: “Zero shows no pain, then as it goes up, it keeps showing a little pain, then a little bit more, then at the end it shows a lot of pain, and ten shows the most pain” (Subject 9; 9 year-old boy with nephrotic syndrome). Eleven of 15 participants correctly identified zero as the number they would choose if they were not experiencing any pain. Three participants incorrectly chose 1. One participant was confused by the question and did not answer. Fourteen of 15 participants correctly identified 10 as the number they would choose if they were in the worst pain of their lives, while one switched his answer from 1 to 10.

#### Ease of use

Thirteen of 14 participants felt that the scale was easy to understand with no changes needed, while one pointed out that the understandability of the scale depends on how the scale is explained to the patient. Seven out of nine participants thought there was nothing confusing about the scale. One said, “[The scale] only shows pain” (Subject 7; 35 year-old man with connective tissue disease), while another asked, “Does it mean the pain only reaches 10, or can it be above 10?” (Subject 10).

The cognitive interviewing process supported that the NRS is also acceptable to patients as a mechanism for assessing pain and represents their pain appropriately.

## Discussion

This study demonstrated the face validity and acceptability of the Swahili versions of the FPS-R and the NRS as single-item pain assessment tools for use in hospitalized Kenyan patients. Of the unidimensional pain assessment tools available, these scales were judged by our team to be most practical for use in our setting in western Kenya, and they also correspond to the Kenyan Hospice and Palliative Care Association’s recommendation of using a numerical scale and a faces scale for pain assessment. These tested translations use straightforward, non-idiomatic Swahili phrasing, which should make them usable in other Swahili-speaking countries of East Africa, as well as in immigrant populations worldwide.

By using comparisons of their current pain to their previous levels of pain as well as to the facial expressions of the FPS-R to select an answer, participants demonstrated rational decision-making processes in choosing responses to the FPS-R and the NRS. They showed good comprehension of the progression from the left-most anchor of no pain to the right-most anchor of the worst pain on both scales. Participants also found the scales easy to use and denied any confusion with the phrasing of instructions or the scales themselves. This comprehension and acceptability to patients is particularly important to demonstrate across cultures, especially in a setting where pain assessment is not routinely done.

There were a few suggestions for changes that could be made to the scales, though none of these were judged to qualify for revision, as they were determined to be subjective preferences (one boy wanted to put ears on the faces) or misunderstandings of the cognitive interview probes (one woman wanted to give the faces medicine to make them feel better). The outcomes of cognitive interviewing help define “tradeoffs” – the advantages and disadvantages of asking a question in a certain manner – rather than dictating a “correct” way to ask a question [35]. In our case, the data generated from cognitive interviewing demonstrated the advantages of keeping the questions as they stand rather than making any changes.

Our team had been worried that the FPS-R would not be understood by Kenyan patients because they might be unfamiliar with “cartoon” faces. There was also a concern that, in this cultural context, patients would be too stoic or too afraid of being considered “weak” to admit to feeling pain. Given the wide range of pain ratings on both scales, including a considerable amount of high scores, and the overwhelmingly favorable acceptance of the faces scale, it seems that these concerns, while still possibly valid, were not the determining factors in our sample. Through the detailed process of cognitive interviewing, Kenyan patients demonstrated understanding and acceptance of both pain scales.

Participants both objectively and subjectively had more difficulties with the NRS than the FPS-R. Our finding of the effectively unanimous preference for the faces scale runs counter to the results of previous studies that have found that older children and adolescents generally prefer a numerical scale or a visual analogue scale to the faces scale [26]. In our study, children and adults across all age ranges preferred the FPS-R. This may be attributable to lower levels of education and difficulties with numeracy. In 2006, Kenya’s adult literacy rate was 61.5%, and its adult numeracy rate was 64.5% [40]. This may have implications for pain scales chosen for use in other populations with low educational levels. Alternatively, our findings may also be due to a cultural preference among this patient population. The subjects raised several questions about the NRS and had more difficulty correctly identifying the number that denotes “no pain,” suggesting that the NRS may be a more problematic scale for use in this population than the FPS-R.

These findings may serve to caution the recent movement towards an international standard for pain assessment in palliative care [21,41]. Based on systematic literature reviews and expert opinion surveys, the NRS has emerged as the lead candidate for universal adoption [21]. While we agree that establishing a universal standard for pain intensity measurement is desirable, it may not be feasible to dictate the use of a single tool that is optimized for all populations. In concert with other studies in non-Caucasian patient groups that have found a preference for the FPS-R, our results serve as a reminder that any efforts to establish a consensus must include representation from non-Western populations [42,43].

Despite the concerns encountered during the cognitive interviewing process, the NRS still has strong potential for clinical use in our population. All the participants were able to choose a response to the NRS, and the FPS-R and NRS ratings were largely consistent subject-by-subject. Previous studies have reported the many advantages of the NRS, including ease of administration, straightforward scoring mechanism, patient preference, and ability to be used in parametric analyses [15,21,44,45]. The NRS and FPS-R have different strengths, and these distinctions can help direct their use in this population in the future. For example, the FPS-R may be more suitable for day-to-day patient care, since subjects preferred this scale and found it easier to understand. On the other hand, the NRS may be more appropriate for research purposes, as it lends itself to statistical analysis and is becoming the international standard, thereby facilitating comparisons to be made with other populations.

There were some potential limitations to this study. We performed 15 cognitive interviews, and if we had conducted more, we may have found more subtle deficiencies in the pain scales. However, experts generally recommend doing a maximum of 12 to 15 cognitive interviews before analysis, as subsequent interviews reach a point of diminishing returns [35]. Additionally, the cognitive interviewers are all part of the hospital’s palliative care team and are familiar with the topics of pain assessment and treatment. While this may have biased them towards overestimation of pain or may have led to a preconceived belief that the pain assessment tools are acceptable and needed, the use of standardized scripts for the cognitive interviewing process, and the process of translation and transcription of the interviews by a non-palliative care team linguist minimizes the influence of interviewer bias on our results.

## Conclusions

In this study, we adapted and translated the FPS-R and NRS for a population of Swahili-speaking patients in western Kenya, and demonstrated the face validity, acceptability, and field-readiness of these scales through cognitive interviewing of hospitalized patients at MTRH. Dissemination and use of these pain tools in Kenya and East Africa could result in increased awareness of patients’ pain and in an appropriate response in relieving their suffering.

## Competing interests

The authors declare that they have no competing interests.

## Authors’ contributions

All authors participated in the design of the study. MH helped with data acquisition. KH, RV, CO, and GPG were involved in data analysis and interpretation. KH, RMS, RV, and GPG were involved in drafting the manuscript. All authors read and approved the final manuscript.

## Authors’ information

KH is a medical student at Harvard Medical School and recently completed a research fellowship at the Academic Model Providing Access to Healthcare (AMPATH) in Eldoret, Kenya. CO is a Lecturer in the Department of Surgery at Moi University School of Medicine in Eldoret, Kenya, where he helped develop a new palliative care curriculum, and an ophthalmology consultant at Moi Teaching and Referral Hospital (MTRH) in Eldoret, Kenya. RCV is an Assistant Professor of Pediatrics at Indiana University School of Medicine in the divisions of Children’s Health Services Research and Pediatric Infectious Diseases and Co-Director of Pediatric Research for AMPATH in Eldoret, Kenya. MH is a medical officer at AMPATH-Oncology and helped develop the standard operating procedures for the palliative care team. FN is a pediatric oncology consultant at MTRH. RMS is an Assistant Professor of Clinical Pharmacology and Oncology at Indiana University School of Medicine and past Co-Director of AMPATH-Oncology in Eldoret, Kenya. GPG is a Professor of Medicine at Indiana University School of Medicine where he directs the Palliative Medicine Fellowship and the Palliative Care Program at Wishard Hospital in Indianapolis, Indiana.

## Pre-publication history

The pre-publication history for this paper can be accessed here:

http://www.biomedcentral.com/1472-684X/11/5/prepub

## Supplementary Material

Additional file 1 Cognitive Interview Probes. Click here for file
