# Supplementary material for: Assessment of the face validity of two pain scales in Kenya: a validation study using cognitive interviewing
Source: BMC Palliat Care. 2012 Jul 10;11:5. doi: 10.1186/1472-684X-11-5 (PMC3393614; doi:10.1186/1472-684X-11-5)
Supplement: Additional file 1 — Cognitive Interview Probes. [file 1472-684X-11-5-S1.doc]

**Appendix A:** Cognitive Interview Probes

**Faces Pain Scale**

These faces show how much something can hurt. The face on the left shows no pain. The faces show more and more pain proceeding from left to right, up to the face on the right – it shows the most pain. Point to the face that shows how much you hurt right now.

- What do you think of this question?
- How did you decide your answer to this question?
- How easy or difficult was it to choose an answer?
- In this question, what does the word “pain” mean to you?
- Look at the faces. What does the expression on each face mean to you?
- Do you think facial expressions are a fair reflection of pain?
- Which face would you choose if you were not experiencing any pain?
- Which face would you choose if you were experiencing the worst pain in your life?
- What would make the instructions or the facial expressions easier to understand?
- Are there any confusing things about this pain scale?
- What questions do you have about this pain scale?

**Numerical Rating Scale**

If 0 means “no pain,” and 10 means “the worst pain that you can imagine,” on this scale from 0 to 10, what is your current level of pain?

**
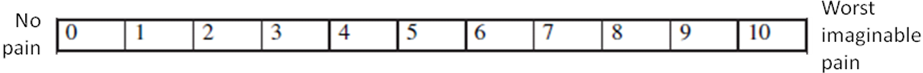
**

- What do you think of this question?
- How did you decide your answer to this question?
- How easy or difficult was it to choose an answer?
- In this question, what does the word “pain” mean to you?
- What do the numbers mean to you?
- Which number would you choose if you were not experiencing any pain?
- Which number would you choose if you were experiencing the worst pain in your life?
- What would make the instructions easier to understand?
- Are there any confusing things about this pain scale?
- What questions do you have about this pain scale?
